# Supplementary material for: MetaSpot: A General Approach for Recognizing the Reactive Atoms Undergoing Metabolic Reactions Based on the MetaQSAR Database
Source: Int J Mol Sci. 2023 Jul 4;24(13):11064. doi: 10.3390/ijms241311064 (PMC10341931; doi:10.3390/ijms241311064)
Supplement: Supplementary file 1 [file ijms-24-11064-s001.zip › ijms-2454440-supplementary tables.pdf]

Article

MetaSpot: a general approach for recognizing the reactive atoms undergoing metabolic reactions based on the MetaQSAR database

Angelica Mazzolari, Pietro Perazzoni, Emanuela Sabato, Filippo Lunghini, Andrea R. Beccari, Giulio Vistoli and Alessandro Pedretti

SUPPORTING INFORMATION

Table S1: Performance metrics (Accuracy, MCC and AUC values) for the 17 considered classes of metabolic reactions in the first round of predictions. For each metric, the reported mean and range values are derived by repeating 100 times the random undersampling of the NR atoms.

Class ID	Cases	Description	Accuracy		MCC		AUC	
			Mean	Range	Mean	Range	Mean	Range
01	3050	Oxidation of Csp3	0.85	0.005	0.72	0.04	0.87	0.03
02	1798	Oxidation of Csp2 and Csp	0.81	0.008	0.63	0.07	0.83	0.06
03	278	CHOH ↔ C═O → COOH	0.90	0.02	0.80	0.19	0.95	0.06
05	252	Redox reactions of R3N	0.94	0.02	0.89	0.14	0.98	0.05
06	330	Redox reactions of >NH, >NOH, and –N═O	0.93	0.01	0.86	0.12	0.97	0.03
07	198	Redox of quinones or analogues	0.74	0.03	0.48	0.30	0.81	0.16
08	274	Redox of S atoms	0.95	0.01	0.89	0.09	0.98	0.04
**Main class 1**		Redox reactions	**0.87**	**0.01**	**0.75**	**0.14**	**0.91**	**0.06**
11	534	Hydrolysis of esters and lactones	0.96	0.01	0.92	0.09	0.98	0.02
12	258	Hydrolysis of amides, lactams and peptides	0.92	0.02	0.83	0.21	0.97	0.06
14	168	Other hydrolyses	0.73	0.03	0.46	0.33	0.80	0.18
**Main class 2**		Hydrolyses	**0.87**	**0.02**	**0.74**	**0.21**	**0.92**	**0.09**
21	800	O-glucuronidations and glycosylations	0.96	0.005	0.92	0.05	0.96	0.04
22	298	N- and S-glucuronidations and glycosilations	0.94	0.01	0.88	0.15	0.95	0.06
23	244	Sulfonations	0.94	0.02	0.89	0.18	0.97	0.07

24	320	GSH and RSH conjugations	0.86	0.02	0.71	0.14	0.92	0.05
25	140	Acetylations and acylations	0.94	0.02	0.88	0.24	0.98	0.05
26	130	CoASH-ligation plus amino acid conjugations	0.95	0.02	0.90	0.15	0.98	0.04
27	110	Methylations	0.89	0.04	0.78	0.33	0.93	0.13
**Main class 3**		**Conjugations**	**0.93**	**0.02**	**0.85**	**0.18**	**0.96**	**0.06**
**Overall means**			**0.89**	**0.02**	**0.79**	**0.17**	**0.93**	**0.07**

Table S2: Descriptors selected by the feature importance analyses for the models developed for the 17 considered classes of metabolic reactions in the first round of predictions (here and in the following Tables the prefixes Atm and Mol stand for atom- and ligand-based descriptors).

Class ID	Description	Selected Features
01	Oxidation of Csp3	Atm_Broto, Atm_Kier, Mol_HbDon
02	Oxidation of Csp2 and Csp	Atm_Kier, Mol_HbDon
03	CHOH ↔ C═O → COOH	Atm_Broto, Atm_Kier, Atm_Charge, Atm_q(r)-Z(r), Atm_Lumo+1, Mol_Lipole
05	Redox reactions of R3N	Atm_Broto, Atm_Dn(r), Atm_Homo-1, Mol_Rotors
06	Redox reactions of >NH, >NOH, and –N═O	Atm_Kier, Atm_piS(r), Atm_Homo-1, Atm_Lumo+1, Mol_Atoms, Mol_HeavyAtoms, Mol_Ovality, Mol_PSA
07	Redox of quinones or analogues	Atm_Broto, Atm_piS(r), Mol_HbAcc, Mol_HbDon, Mol_Impropers, Mol_Surface
08	Redox of S atoms	Atm_Broto, Atm_Kier, Atm_piS(r), Mol_Gyrrad, Mol_Lipole
11	Hydrolysis of esters and lactones	Atm_Broto, Atm_Kier, Atm_Charge, Atm_Dn(r), Mol_Dipole, Mol_Impropers, Mol_Sas
12	Hydrolysis of amides, lactams and peptides	Atm_Broto, Atm_Kier, Atm_piS(r), Mol_Rotors, Mol_Gyrrad, Mol_LogP
14	Other hydrolyses	Atm_Kier, Atm_q(r)-Z(r), Atm_piS(r), Atm_Lumo, Mol_Gyrrad, Mol_HbDon
21	O-glucuronidations and glycosylations	Atm_Kier
22	N-/S-glucuronidations/glycosilations	Atm_Broto
23	Sulfonations	Atm_Kier, Mol_HbAcc
24	GSH and RSH conjugations	Atm_Broto, Atm_Charge, Atm_piS(r), Mol_Impropers
25	Acetylations and acylations	Atm_Kier, Atm_piS(r), Mol_Impropers
26	CoASH-ligation plus amino acid conjugations	Atm_Broto, Atm_Kier, Atm_Charge, Atm_piS(r), Atm_Lumo+1, Mol_Gyrrad, Mol_HbAcc, Mol_HbDon, Mol_Impropers, Mol_LogP, Mol_Volume
27	Methylations	Atm_Broto, Atm_q(r)-Z(r), Mol_Rotors, Mol_HbAcc, Mol_HeavyAtoms, Mol_Mass

Table S3: Performance metrics (Accuracy, MCC and AUC values) for the 17 considered classes of metabolic reactions as derived by the second round of predictions in which the NR atoms are purposely selected to have the same atom type(s) of the reactive centers. The differences compared to the first round in accuracy and MCC values are also reported.

Class ID	Description	Accuracy	MCC	AUC	ΔMCC	ΔAccuracy
01	Oxidation of Csp3	0.87	0.75	0.93	+0.03	+0.03
02	Oxidation of Csp2 and Csp	0.90	0.79	0.93	+0.16	+0.09
03	CHOH ↔ C=O → COOH	0.83	0.65	0.89	-0.15	-0.04
05	Redox reactions of R3N	0.71	0.41	0.78	-0.48	-0.19
06	Redox reactions of >NH, >NOH, and –N=O	0.69	0.38	0.75	-0.48	-0.25
07	Redox of quinones or analogues	0.80	0.60	0.91	+0.12	+0.06
08	Redox of S atoms	0.83	0.59	0.87	-0.30	-0.09
**Main class 1**	Redox reactions	**0.80**	**0.60**	**0.86**	**-0.16**	**-0.06**
11	Hydrolysis of esters and lactones	0.74	0.47	0.83	-0.45	-0.22
12	Hydrolysis of amides, lactams and peptides	0.78	0.55	0.83	-0.28	-0.14
14	Other hydrolyses	0.76	0.52	0.85	+0.06	+0.03
**Main class 2**	Hydrolyses	**0.76**	**0.51**	**0.84**	**-0.22**	**-0.11**
21	O-glucuronidations and glycosylations	0.58	0.15	0.61	-0.77	-0.38
22	N-/S-glucuronidations/glycosilations	0.54	0.07	0.59	-0.81	-0.40
23	Sulfonations	0.71	0.42	0.75	-0.47	-0.23
24	GSH and RSH conjugations	0.78	0.56	0.89	-0.15	-0.08
25	Acetylations and acylations	0.73	0.46	0.79	-0.42	-0.22
26	CoASH-ligation plus amino acid conjugations	0.88	0.77	0.96	-0.13	-0.07
27	Methylations	0.76	0.52	0.81	-0.26	-0.13
**Main class 3**	**Conjugations**	**0.71**	**0.42**	**0.77**	**-0.43**	**-0.22**
**Overall means**		**0.76**	**0.51**	**0.82**	**-0.27**	**-0.13**

Table S4: Descriptors selected by the feature importance analyses for the models developed for the 17 considered classes of metabolic reactions in the first round of predictions.

Class ID	Description	Selected Features
01	Oxidation of Csp3	Atm_piS(r), Mol_Dipole, Mol_Lipole, Mol_Mass, Mol_Ovality, Mol_PSA, Mol_LogP, Mol_Volume
02	Oxidation of Csp2 and Csp	Atm_Charge, Atm_piS(r), Mol_Impropers, Mol_PSA, Mol_LogP
03	CHOH ↔ C=O → COOH	Atm_Charge, Atm_q(r)-Z(r), Mol_Lipole, Mol_Mass, Mol_PSA
05	Redox reactions of R3N	Atm_Charge, Atm_piS(r), Mol_Dipole, Mol_Surface
06	Redox reactions of >NH, >NOH, and –N=O	Atm_Charge, Atm_Homo-1, Mol_HbAcc, Mol_Impropers, Mol_PSA
07	Redox of quinones or analogues	Atm_Homo-1, Atm_Lumo, Mol_Atoms, Mol_Dipole, Mol_HbDon, Mol_Sav
08	Redox of S atoms	Atm_Dn(r), Atm_q(r)-Z(r), Atm_Homo, Mol_HbDon, Mol_PSA
11	Hydrolysis of esters and lactones	Atm_Charge, Atm_piS(r), Atm_Homo-1, Mol_Impropers, Mol_Mass, Mol_PSA
12	Hydrolysis of amides, lactams and peptides	Atm_piS(r), Mol_Gyrrad, Mol_Impropers, Mol_PSA
14	Other hydrolyses	Mol_Gyrrad, Mol_Impropers, Mol_Lipole, Mol_PSA, Mol_LogP
21	O-glucuronidations and glycosylations	Atm_De(r), Mol_HbDon
22	N-/S-glucuronidations/glycosilations	Mol_Atoms, Mol_Dipole, Mol_LogP
23	Sulfonations	Atm_Dn(r), Atm_Homo, Mol_Rotors, Mol_Gyrrad, Mol_HbDon, Mol_PSA
24	GSH and RSH conjugations	Atm_Dn(r), Atm_Lumo, Mol_Atoms, Mol_Gyrrad
25	Acetylations and acylations	Atm_Charge, Atm_Dn(r), Atm_Lumo, Mol_PSA
26	CoASH-ligation plus amino acid conjugations	Atm_Charge, Atm_piS(r), Atm_Lumo+1
27	Methylations	Mol_Atoms, Mol_Rotors, Mol_Gyrrad, Mol_HbAcc, Mol_Lipole

Table S5: Performance metrics (Accuracy, MCC and AUC values) for the considered subclasses of metabolic reactions. For each metric, the mean and range values are derived by repeating 100 times the random undersampling of the NR atoms.

Subclass ID	Description	Cases	Accuracy		MCC		AUC	
			Mean	Range	Mean	Range	Mean	Range
01.01	Oxidations of isolated Csp3	193	0.84	0.08	0.70	0.03	0.90	0.06
01.02	Oxidations of C in α to an unsaturated system	233	0.92	0.05	0.84	0.10	0.96	0.04
01.03	Oxidations of Csp3 carrying an heteroatom	492	0.90	0.04	0.81	0.09	0.94	0.04
01.04	Dehydrogenations	81	0.79	0.14	0.58	0.27	0.83	0.14
02.01	Oxidations of aryl compounds	440	0.82	0.04	0.63	0.08	0.82	0.07
02.02	Oxidations of azarenes	94	0.88	0.13	0.77	0.26	0.93	0.10
02.03	Oxidations of >C=C<	55	0.90	0.13	0.79	0.26	0.94	0.09
03.02	Hydrogenations of carbonyls	71	0.95	0.09	0.89	0.18	0.97	0.05
05.01	Oxidations of tertiary alkylamines	65	0.95	0.08	0.91	0.16	0.99	0.04
06.01	Hydroxylations of amines	62	0.98	0.05	0.96	0.11	0.99	0.01
07.04	Oxidations of phenols	51	0.73	0.25	0.45	0.50	0.79	0.26
08.03	Oxygenations of sulfides	83	0.99	0.05	0.97	0.08	0.99	0.02
**Main class 1**	**Average**		**0.89**	**0.09**	**0.78**	**0.18**	**0.92**	**0.08**
11.01	Hydrolysis of alkyl esters	103	0.98	0.06	0.95	0.11	0.99	0.05
11.03	Hydrolysis of anionic and cationic esters	100	0.97	0.07	0.93	0.13	0.98	0.06
11.08	Hydrolysis of esters of inorganic acids	51	0.91	0.18	0.81	0.35	0.97	0.08
12.02	Hydrolysis of anilides and hydrazides	55	0.93	0.13	0.85	0.26	0.97	0.08
**Main class 2**	**Average**		**0.94**	**0.11**	**0.88**	**0.21**	**0.98**	**0.07**
21.01	O-glucuronidation of alcohols	85	0.97	0.05	0.94	0.11	0.97	0.07
21.02	O-glucuronidation of phenols	152	0.94	0.05	0.92	0.10	0.98	0.04
21.03	O-glucuronidation of carboxylic acids	99	0.96	0.08	0.92	0.16	0.99	0.03
22.01	N-glucuronidation of linear and cyclic amines	97	0.95	0.09	0.90	0.19	0.97	0.07
23.01	O-sulfonation of phenols	70	0.94	0.17	0.88	0.34	0.97	0.08
24.01	Nucleophilic additions of glutathione	89	0.88	0.11	0.76	0.23	0.94	0.07
24.02	Reactions of glutathione addition-elimination	68	0.81	0.13	0.61	0.29	0.77	0.19
**Main class 3**	**Average**		**0.92**	**0.10**	**0.85**	**0.20**	**0.94**	**0.08**

Table S6: Descriptors selected by the feature importance analyses for the models developed for the considered subclasses of metabolic reactions in the first round of predictions.

Subclass ID	Description	Accuracy
01.01	Oxidations of isolated Csp3	Atm_Broto, Atm_Kier, Atm_Charge, Atm_Dn(r), Atm_piS(r), Atm_Homo, Atm_Lumo+1, Mol_Atoms, Mol_Impropers
01.02	Oxidations of C in α to an unsaturated system	Atm_Broto, Atm_Charge, Atm_piS(r), Mol_Rotors, Mol_HbDon
01.03	Oxidations of Csp3 carrying an heteroatom	Atm_Kier, Atm_Charge, Atm_piS(r), Atm_Homo-1, Mol_Atoms, Mol_HbAcc, Mol_HeavyAtoms, Mol_Mass
01.04	Dehydrogenations	Atm_Broto, Atm_Charge, Atm_q(r)-Z(r), Atm_piS(r), Atm_Lumo+1, Mol_HbDon, Mol_Mass, Mol_Volume
02.01	Oxidations of aryl compounds	Atm_Broto, Mol_HbDon
02.02	Oxidations of azarenes	Atm_Broto, Atm_Kier, Atm_Charge, Atm_Dn(r), Atm_De(r), Atm_piS(r), Atm_Homo-1, Mol_Dipole, Mol_Sav
02.03	Oxidations of >C=C<	Atm_Broto, Atm_Kier, Atm_Dn(r), Atm_piS(r), Atm_Homo-1, Mol_Rotors
03.02	Hydrogenations of carbonyls	Atm_Broto, Atm_Kier, Atm_Charge, Atm_De(r), Mol_Lipole
05.01	Oxidations of tertiary alkylamines	Atm_Kier, Atm_Homo-1, Mol_Dipole, Mol_Rotors, Mol_HbAcc, Mol_PSA
06.01	Hydroxylations of amines	Atm_Broto, Atm_Kier, Atm_Charge
07.04	Oxidations of phenols	Atm_Broto, Atm_Kier, Atm_Charge, Mol_HeavyAtoms, Mol_Volume
08.03	Oxygenations of sulfides	Atm_Broto, Atm_Kier, Atm_Charge, Atm_piS(r), Atm_Homo, Mol_Atoms, Mol_HbAcc
11.01	Hydrolysis of alkyl esters	Atm_Kier, Atm_Charge, Mol_HbAcc
11.03	Hydrolysis of anionic and cationic esters	Atm_Broto, Atm_Charge, Mol_Dipole
11.08	Hydrolysis of esters of inorganic acids	Atm_Kier, Atm_Lumo, Atm_Lumo+1, Mol_Atoms, Mol_Lipole
12.02	Hydrolysis of anilides and hydrazides	Atm_Broto, Atm_Charge, Atm_q(r)-Z(r)
21.01	O-glucuronidation of alcohols	Atm_Broto, Atm_Kier
21.02	O-glucuronidation of phenols	Atm_Broto, Atm_Kier, Mol_HbAcc, Mol_HbDon
21.03	O-glucuronidation of carboxylic acids	Atm_Broto, Atm_Kier, Atm_Homo
22.01	N-glucuronidation of linear and cyclic amines	Atm_Kier, Atm_Charge, Atm_piS(r), Atm_Homo, Atm_Lumo+1, Mol_Dipole, Mol_Rotors, Mol_HeavyAtoms, Mol_Impropers
23.01	O-sulfonation of phenols	Atm_Broto, Atm_Kier, Atm_q(r)-Z(r), Mol_Gyrrad
24.01	Nucleophilic additions of glutathione	Atm_Broto, Atm_Kier, Atm_Charge, Atm_piS(r), Atm_Lumo+1, Mol_Impropers
24.02	Reactions of glutathione addition−elimination	Atm_Broto

Table S7: Performance metrics (Accuracy, MCC and AUC values) for the considered subclasses of metabolic reactions as derived by the second round of predictions in which the NR atoms are purposely selected to have the same atom type(s) of the reactive centers. The differences with the first round in accuracy and MCC values are also reported.

Subclass ID	Description	Accuracy	MCC	AUC	ΔAccuracy	ΔMCC
01.01	Oxidations of isolated Csp3	0.87	0.73	0.93	+0.03	+0.02
01.02	Oxidations of C in α to an unsaturated system	0.85	0.68	0.92	-0.07	-0.16
01.03	Oxidations of Csp3 carrying an heteroatom	0.86	0.71	0.90	-0.04	-0.10
01.04	Dehydrogenations	0.82	0.65	0.88	+0.03	+0.07
02.01	Oxidations of aryl compounds	0.88	0.75	0.92	+0.06	+0.12
02.02	Oxidations of azarenes	0.91	0.82	0.97	+0.03	+0.07
02.03	Oxidations of >C=C<	0.75	0.50	0.84	-0.15	-0.29
03.02	Hydrogenations of carbonyls	0.75	0.50	0.82	-0.20	-0.39
05.01	Oxidations of tertiary alkylamines	0.72	0.43	0.82	-0.15	-0.49
06.01	Hydroxylations of amines	0.93	0.87	0.98	-0.05	-0.09
07.04	Oxidations of phenols	0.82	0.66	0.85	+0.09	+0.19
08.03	Oxygenations of sulfides	0.84	0.69	0.84	-0.15	-0.27
**Main class 1**	**Average**	**0.83**	**0.66**	**0.89**	**-0.06**	**-0.11**
11.01	Hydrolysis of alkyl esters	0.73	0.46	0.84	-0.25	-0.49
11.03	Hydrolysis of anionic and cationic esters	0.76	0.52	0.89	-0.21	-0.41
11.08	Hydrolysis of esters of inorganic acids	0.80	0.60	0.83	-0.11	-0.21
12.02	Hydrolysis of anilides and hydrazides	0.72	0.44	0.79	-0.21	-0.41
**Main class 2**	**Average**	**0.75**	**0.51**	**0.84**	**-0.19**	**-0.38**
21.01	O-glucuronidation of alcohols	0.58	0.16	0.63	-0.39	-0.77
21.02	O-glucuronidation of phenols	0.71	0.43	0.77	-0.23	-0.49
21.03	O-glucuronidation of carboxylic acids	0.89	0.77	0.94	-0.07	-0.15
22.01	N-glucuronidation of linear and cyclic amines	0.58	0.17	0.65	-0.37	-0.73
23.01	O-sulfonation of phenols	0.72	0.44	0.80	-0.22	-0.44
24.01	Nucleophilic additions of glutathione	0.92	0.84	0.97	+0.04	+0.08
24.02	Reactions of glutathione addition–elimination	0.78	0.55	0.84	-0.03	-0.06
**Main class 3**	**Average**	**0.74**	**0.48**	**0.80**	**-0.18**	**-0.36**

Table S8: Descriptors selected by the feature importance analyses for the models developed for the considered subclasses of metabolic reactions in the second round of predictions.

Subclass ID	Description	Accuracy
01.01	Oxidations of isolated Csp3	Atm_piS(r), Mol_Dipole, Mol_Lipole, Mol_Mass, Mol_PSA, Mol_LogP
01.02	Oxidations of C in α to an unsaturated system	Atm_Charge, Atm_piS(r), Mol_Mass
01.03	Oxidations of Csp3 carrying an heteroatom	Atm_Charge, Atm_q(r)-Z(r), Mol_PSA, Mol_LogP, Mol_Volume
01.04	Dehydrogenations	Atm_piS(r), Atm_q(r)-Z(r), Mol_Dipole, Mol_Rotors, Mol_HeavyAtoms, Mol_Impropers, Mol_Lipole, Mol_PSA
02.01	Oxidations of aryl compounds	Atm_Charge, Atm_Dn(r), Atm_Homo, Mol_Dipole, Mol_Gyrrad, Mol_Lipole, Mol_Mass, Mol_PSA Mol_LogP, Mol_Volume
02.02	Oxidations of azarenes	Atm_Charge, Atm_q(r)-Z(r), Atm_piS(r), Mol_Gyrrad, Mol_HbAccì, Mol_LogP
02.03	Oxidations of >C=C<	Atm_Dn(r), Atm_Homo-1, Atm_Homo, Atm_Lumo, Mol_Gyrrad, Mol_LogP
03.02	Hydrogenations of carbonyls	Atm_Charge, Atm_q(r)-Z(r), Atm_Lumo+1, Mol_HbAcc, Mol_PSA
05.01	Oxidations of tertiary alkylamines	Atm_Charge, Atm_De(r), Atm_piS(r), Mol_Gyrrad
06.01	Hydroxylations of amines	Atm_De(r), Atm_piS(r), Mol_PSA
07.04	Oxidations of phenols	Atm_q(r)-Z(r), Atm_piS(r), Mol_Atoms, Mol_Dipole, Mol_LogP
08.03	Oxygenations of sulfides	Atm_Charge, Atm_q(r)-Z(r), Atm_Homo, Atm_Lumo, Mol_HbDon
11.01	Hydrolysis of alkyl esters	Atm_Charge, Atm_piS(r), Atm_Homo-1, Mol_Impropers, Mol_Sas
11.03	Hydrolysis of anionic and cationic esters	Atm_piS(r), Mol_Impropers
11.08	Hydrolysis of esters of inorganic acids	Atm_Charge, Atm_Dn(r), Atm_q(r)-Z(r), Atm_Lumo+1
12.02	Hydrolysis of anilides and hydrazides	Atm_Charge, Atm_piS(r), Mol_HeavyAtoms, Mol_Impropers
21.01	O-glucuronidation of alcohols	Atm_Charge, Atm_Dn(r), Mol_HbDon, Mol_Impropers, Mol_Sav
21.02	O-glucuronidation of phenols	Atm_Charge, Atm_Dn(r), Atm_De(r), Atm_q(r)-Z(r), Atm_Homo, Mol_Gyrrad, Mol_HbDon, Mol_Volume, Mol_HeavyAtoms, Mol_PSA, Mol_LogP,
21.03	O-glucuronidation of carboxylic acids	Atm_Charge, Atm_piS(r), Atm_Homo-1
22.01	N-glucuronidation of linear and cyclic amines	Atm_Charge, Atm_piS(r), Mol_Gyrrad, Mol_Impropers, Mol_PSA
23.01	O-sulfonation of phenols	Atm_Charge, Atm_De(r), Atm_Homo, Mol_Gyrrad, Mol_HbDon, Mol_Lipole, Mol_PSA, Mol_Sas
24.01	Nucleophilic additions of glutathione	Atm_Charge, Atm_Dn(r), Atm_piS(r), Atm_Lumo Mol_Rotors, Mol_Impropers, Mol_Ovality
24.02	Reactions of glutathione addition–elimination	Atm_Lumo, Atm_Lumo+1, Mol_Atoms, Mol_Dipole Mol_Gyrrad, Mol_LogP
